# Impact of face masks and sunglasses on attractiveness, trustworthiness, and familiarity, and limited time effect: a Japanese sample

**DOI:** 10.1007/s44202-023-00066-6

**Published:** 2023-01-24

**Authors:** Takuma Takehara, Mahiro Kaigawa, Aika Kobayashi, Yuuka Yamaguchi

**Affiliations:** Department of Psychology, Doshisha University, 1-3 Tatara-Miyakodani, Kyotanabe, Kyoto 610-0394 Japan

**Keywords:** Face masks, Sunglasses, Time effect, Attractiveness, Trustworthiness, Familiarity

## Abstract

Many studies conducted after the COVID-19 pandemic have examined the relationship between changes in social traits, such as attractiveness and wearing face masks. However, most studies examine the effect of wearing face masks at a single time point, and the time effect is not known. Additionally, few studies address wearing sunglasses, another facial occluding item. This study examined the effects of facial occluding (unoccluded face, face masks, sunglasses, or both) on perceived attractiveness, trustworthiness, and familiarity at two time points, September 2020, six months after the start of the COVID-19 pandemic, and April 2022, almost two years later, using Japanese higher and lower attractive faces. Results showed that only lower attractive faces wearing face masks had a time effect on attractiveness and familiarity and no time effect on social traits in higher attractive faces. Perceived all social traits were the highest for unoccluded faces, and faces wearing face masks had the same level of attractiveness and familiarity as unoccluded faces. Perceived trustworthiness was higher for unoccluded faces, faces wearing face masks, sunglasses, and both sunglasses and face masks, respectively. Additionally, faces wearing both sunglasses and face masks had the lowest perceived all social traits. These findings suggest that the positive and time effects of wearing face masks are limited in Japan, suggesting a greater positive impact of unoccluded faces. They also suggest that the negative impact of wearing sunglasses is significant.

## Introduction

Human faces convey several social traits in social communication, including emotion, personality, age, attractiveness, trustworthiness, and familiarity. In particular, facial attractiveness influences various social situations [1], and compared to people with low attractive faces (LAFs), people with high attractive faces (HAFs) benefit in various ways [2]. Furthermore, people with HAFs are perceived as having socially and morally desirable personalities [3–5]. This is referred to as the "beauty-is-good" bias. There is a positive correlation between perceived attractiveness and trustworthiness, and people with HAF are judged to be more trustworthy [6–9]. Correlations with attractiveness have also been found in social traits such as warmth [10] and approachableness [7].

Items worn on the face can easily and noninvasively alter the social traits conveyed by the face. To the best of our knowledge, the only study that has examined the effects of face masks on social traits perceived from the face before the COVID-19 pandemic is the study by Miyazaki and Kawahara [11]. Their prominent study examined the effects of facial masks on perceived attractiveness and healthiness. The results showed that when people with HAFs wore face masks, their perceived attractiveness was lower than when they did not wear them, but this effect did not occur in people with LAFs. Additionally, regardless of the level of attractiveness, faces wearing face masks were judged to be unhealthier than those not wearing them. They called these phenomena the sanitary mask effect.

After the COVID-19 pandemic declared by the World Health Organization (WHO) on March 11, 2020, the use of face masks became mandatory for reducing the public health burden of COVID-19 infection [12, 13]. Around the same time, the association between wearing face masks and perceived social traits began to be examined by many researchers worldwide. For example, Kamatani et al. [14] conducted a similar experiment to Miyazaki and Kawahara [11] again after the COVID-19 pandemic. They found that perceived attractiveness increased when people with LAFs wore face masks and conversely decreased perceived attractiveness for people with HAFs, suggesting partial inconsistency with the results reported by Miyazaki and Kawahara [11]. Pazhoohi and Kingston [15] found that perceived attractiveness increased when people with LAFs wore face masks, but no effect was found in people with HAFs, indicating disagreement with Miyazaki and Kawahara’s [11] results. Patel et al. [16] also examined the association between wearing face masks and perceived attractiveness. The results showed that perceived attractiveness increased when wearing face masks for both people with LAFs and HAFs, suggesting inconsistency with Miyazaki and Kawahara [11].

However, several studies on perceived trustworthiness have been reported. Gabrieli and Esposito [17] conducted an experiment between August and September 2021 after the COVID-19 pandemic and found that faces wearing face masks were less trustworthy compared to unoccluded faces. They argued that when a face mask partially covered a face, context cues used to predict attributes were invisible, establishing a sense of insecurity that impacts social judgments. Contrary to their research, however, several studies have reported that faces wearing face masks were perceived as more trustworthy than unoccluded faces [18–20]. Guo et al. [18] argued that mask-wearers are likely to be viewed as those who are more considerate, law-binding, and rule followers, care for their own health, and are less harmful to others. Similarly, Olivera-La Rosa et al. [20] also found that faces wearing face masks were perceived as more trustworthy than unoccluded faces, suggesting that people with high social trust have a positive bias towards most people, which in turn promotes social contact.

Several studies have examined the effects of wearing face masks on so-called familiarity, such as approachability, proximity, friendliness, and closeness. For example, it was found that people wearing face masks were perceived as more approachable [18, 19] and proximitous [21], compared to people not wearing face masks. According to Guo et al. [18], approachability is positively correlated with trustworthiness, and this mask-enhanced approachableness could be due to current practice and social conformity. Additionally, Olivera-La Rosa et al. [20] reported closer social distance for faces wearing face masks compared to unoccluded faces. These studies suggest that wearing face masks may reduce social distancing after the COVID-19 pandemic.

Another item that can alter facial traits is eyewear. A certain amount of research has long been reported on changes in social traits due to spectacles [22–24]. Unlike glasses, sunglasses, which occlude the upper half of the face, including the eye area, block both sunlight and gaze. Surprisingly, few studies have examined the effects of sunglasses on perceived social traits from faces. Studies have shown that faces wearing sunglasses are rated as cool and positive in the selfie images uploaded to social networking sites [25], and that sunglasses increase perceived attractiveness because they enhance facial symmetry [26]. However, in Graham and Ritchie’s famous study [27] that explored the association between wearing sunglasses and perceived social traits, participants were presented with faces without glasses, faces wearing glasses, and faces wearing sunglasses, and asked to rate three social traits (attractiveness, trustworthiness, and competence). Results showed that perceived attractiveness and competence did not change when sunglasses were worn, but trustworthiness decreased. More recently, Bennetts et al. [28] found that compared to unoccluded faces, faces wearing sunglasses and faces wearing both sunglasses and face masks showed a decrease in both perceived attractiveness and trustworthiness. Additionally, there are interesting studies on Airbnb guests’ and hosts’ profile photos. According to Karlsson et al. [29], when Airbnb guests send profile photos wearing sunglasses to their hosts, they are more likely to be denied permission to stay by their hosts. Snehasish et al. [30] found that wearing sunglasses in profile photos of Airbnb hosts decreases perceived trustworthiness and intent to book. Thus, while changes in perceived attractiveness are not constant, perceived trustworthiness decreases uniformly when sunglasses are worn. The reason for this is that the eye area is very important in assessing trustworthiness [31], and a face wearing sunglasses that block the gaze loses a source of trustworthiness information.

The abovementioned studies using sunglasses were conducted on Westerners. Westerners wear sunglasses on a daily basis as a sun-protective behavior. For example, 52.3% of people in Germany reported wearing sunglasses [32], indicating that wearing sunglasses is usual. However, the frequency and purpose of wearing sunglasses in daily life vary by country and culture. Westerners may be surprised to learn that Japanese people rarely wear sunglasses, even in the scorching heat of mid-summer. In fact, an observation of Japanese pedestrians in the center of Tokyo, the capital of Japan, during midday in midsummer reported that less than 10% wore sunglasses [33]. Unlike Europe and the U.S., there is no culture in Japan in which sunglasses are worn on a daily basis, and Japanese people are not familiar with wearing sunglasses.

As reviewed above, an increasing number of studies worldwide explored the effects of wearing face masks on perceived social traits after the COVID-19 pandemic. However, the effects of wearing face masks on perceived attractiveness have been inconsistent across studies, with differences between LAFs and HAFs [14–16] and differences in perceived trustworthiness between faces wearing face masks and unoccluded faces [17–20]. Additionally, although perceived attractiveness in Japanese has been investigated by Kamatani et al. [14], no Japanese data on trustworthiness and familiarity exist. However, although there have been several studies exploring the effects of sunglasses that occlude the upper half of the face, including the eye area, on social traits conveyed from the face before the COVID-19 pandemic [27, 30], to the best of our knowledge, no data from Japanese samples exist. Furthermore, only the previously reviewed study by Bennetts et al. [28] examined the combined effects of facial occluding in the form of face masks and sunglasses.

The effects of occlusion on social traits may change over time [28]. Most studies previously reviewed measured the effect of occlusion at a single time point. Bennetts et al. [28] examined the effects of combinations of face masks and sunglasses on social traits at three time points after the COVID-19 pandemic. The results showed no effect of face masks or sunglasses over time. However, it would be premature to generalize their findings as there are indications that cultural differences may mediate the effects of occluding facial features on social traits [15, 34]. Additionally, Japanese people continue wearing face masks everywhere because of social norms [35], even though they do not have to wear them in places where the risk of infection with COVID-19 is low. As opinions and recognitions of face masks have been suggested to change over time [36, 37], occluding the lower half of the face may cause temporal changes in perceived social traits. Frequent exposure to faces wearing face masks over a long period of time may cause a mere exposure effect [38], which may result in temporal changes in perceived social traits.

## The present study

This study aimed to examine the effects of occluding faces of Japanese models with HAFs and LAFs with face masks and sunglasses, and combinations thereof, on the ratings of social traits (attractiveness, trustworthiness, and familiarity) by Japanese participants over time. The same experiment was conducted with different participants at two time points in this study: late September 2020 (Former), six months after the declaration of the COVID-19 pandemic, and early April 2022 (Latter), almost two years after the pandemic. In Japan, Former was between the second and third waves of the COVID-19 outbreak, with about 500 new cases per day, and Latter was in the middle of the sixth wave, with about 50,000 new cases per day. The number of new cases was two orders of magnitude higher during the Latter period than during the Former period.

We set three hypotheses. The first is the effect of occlusion on perceived attractiveness and the time effect. As reported by Kamatani et al. [14], using a Japanese sample, we predicted that wearing face masks on LAFs would result in higher perceived attractiveness than unoccluded faces in Former and conversely lower in HAFs. Additionally, as Japanese people continue wearing face masks, we expected that the mere exposure effect [38] would increase the attractiveness of both LAFs and HAFs with face masks in Latter more than in Former. As in Bennetts et al. [28], however, we predicted that in Former, for both LAFs and HAFs, faces wearing sunglasses and both sunglasses and face masks would have lower perceived attractiveness than faces wearing face masks or unoccluded faces. Additionally, as Japanese people seldom wear sunglasses [33], mere exposure effects should not occur in faces wearing sunglasses for both LAFs and HAFs in Latter.

The second hypothesis is the effect of occlusion on perceived trustworthiness and time effect. As previously mentioned, several studies have reported that faces wearing face masks have higher perceived trustworthiness than unoccluded faces [18–20]. In terms of the Japanese people, keeping wearing face masks after the COVID-19 pandemic conforms to social norms [35], which in turn leads to trustworthiness. Thus, for both LAFs and HAFs in the experiment in Former, we can predict that faces wearing face masks have higher perceived trustworthiness compared to unoccluded faces. As the Japanese people continue wearing face masks over time, we would also expect that in the Latter experiment, perceived trustworthiness from faces wearing face masks would remain at the same level as in the Former, or even be higher, for both LAFs and HAFs. However, as several studies have reported [27, 28, 30], faces wearing sunglasses and both face masks and sunglasses would have less perceived trustworthiness than unoccluded faces or faces wearing face masks for both LAFs and HAFs in the experiment in Former. Furthermore, this effect would be replicated in the Latter experiment.

The third hypothesis is the effect of occlusion on perceived familiarity and its time effect. Studies after the COVID-19 pandemic show that wearing face masks increases familiarity [18–21]. Thus, we can predict that faces wearing face masks would have higher perceived familiarity compared to unoccluded faces for both LAFs and HAFs in the Former experiment. However, we can predict that both LAFs and HAFs would maintain the same level of perceived familiarity from faces wearing face masks or even increase it in the Latter experiment. Faces wearing sunglasses, and faces wearing both face masks and sunglasses, would have lower perceived familiarity for both LAFs and HAFs compared to unoccluded faces and faces wearing face masks in the Former and Latter experiments.

## Method

### Participants

Based on the number of participants in a directly related previous study [28], we aimed to recruit 50 participants in each of the Former and Latter experiments in this study. For both experiments, participants were recruited through online messages to university students who had registered for classes. Consequently, 149 students in the Former and 127 in the Latter participated in the experiment. All participants were Japanese and they received course credit for taking part. Informed consent was obtained online from all participants prior to both experiments. The two experiments were approved by the IRB of the first author's department (SJ20028 & SJ22001, respectively).

To maintain data integrity, we applied three pre-registered exclusion criteria to the data prior to analysis in the following order. (1) Participants with extremely fast (less than 200 s) or extremely slow (more than 1000 s) task completion times. (2) Participants with a standard deviation of 0.5 or less for either attractiveness, trustworthiness, or familiarity ratings. (3) Participants with an average attractiveness rating of 1.5 or less for HAFs or 4.5 or higher for LAFs, because the participants did not attempt to devote attentional resources to the ratings and were considered to have repeated ratings inappropriately; 77 participants (44 in Former and 33 in Latter) were excluded. Then, to ensure the same number of participants as targeted in the previous study [28], a random function in Microsoft Excel was used on the remaining data (105 in Former and 94 in Latter) to extract a completely random sample of equal numbers of males and females (25 males and 25 females in each of Former and Latter). Finally, the data of 50 participants from the Former experiment (25 males and 25 females, *M*_age_ = 20.00, *SD*_age_ = 0.00) and 50 from the Latter experiment (25 males and 25 females, *M*_age_ = 19.64, *SD*_age_ = 0.82) were used in the analysis.

### Experimental design

A three-factorial mixed-design was employed, with time point (Former and Latter) as a between-participants factor, and base attractiveness of facial stimuli (HAF and LAF) and type of occlusion (no occlusion, face mask, sunglasses, and combination of face mask and sunglasses) as within-participants factors.

### Facial stimuli

**Fig. 1:**
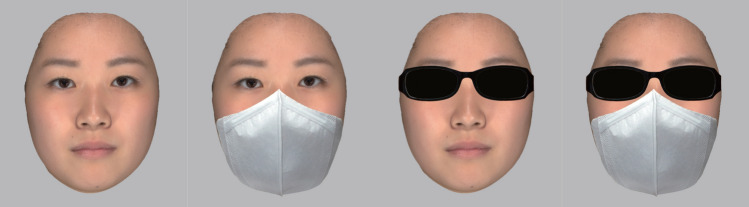
Examples of facial stimuli used in this experiment. From left to right, the presented images illustrate example faces in the no occlusion, face mask, sunglasses, and combination of face mask and sunglasses conditions, respectively. Note that these faces were not actually used in this experiment. In order to protect the privacy of models, these example faces were average faces generated from three models

Before the experiment, the upright and expressionless faces of 85 females who agreed to have their faces photographed and used in the experiment were captured with a digital camera (Canfield Technologies, VECTRA M3). Facial images were cropped for ears and hair using facial image processing software (Medic Engineering, HBM-Rugle), and local cues to the perception of attractiveness, such as blemishes and acne on facial stimuli, were removed using image retouching software (Adobe, Photoshop CS6). Then, 34 university students (17 males and 17 females), who had no relation with the experiment, were asked to rate how attractive they were on a 5-point Likert-type scale (1: not at all attractive to 5: very attractive) using a smartphone or tablet device. Note that we did not measure perceived trustworthiness and familiarity because, as noted at the beginning of the Introduction, a beauty-is-good bias was anticipated. Calculating the average attractiveness scores for each of the 85 facial images, the most attractive face (average of 3.26 points) to the least attractive face (average of 1.29 points) were identified. We selected the top five and the bottom five in attractiveness from these facial images and designated them as HAFs and LAFs, respectively. Ten total facial images of HAFs and LAFs were superimposed with white face masks, dark sunglasses, or both using image retouching software (Adobe, Photoshop CS6). Finally, a total of 40 facial images were prepared: 10 with white face masks, 10 with dark sunglasses, 10 with both, and 10 without any occlusion (Fig. 1).

### Procedure

Laboratory experiments were prohibited by the author's department to prevent the spread of COVID-19 infection. However, research has reported that perceived attractiveness and trustworthiness can be measured by online experiments [39]. Studies have also reported high positive correlations in perceived attractiveness measured in face-to-face and Internet-based experiments [40, 41]. Based on these studies, we determined that an online experiment, rather than a laboratory experiment, was suitable.

**Fig. 2:**
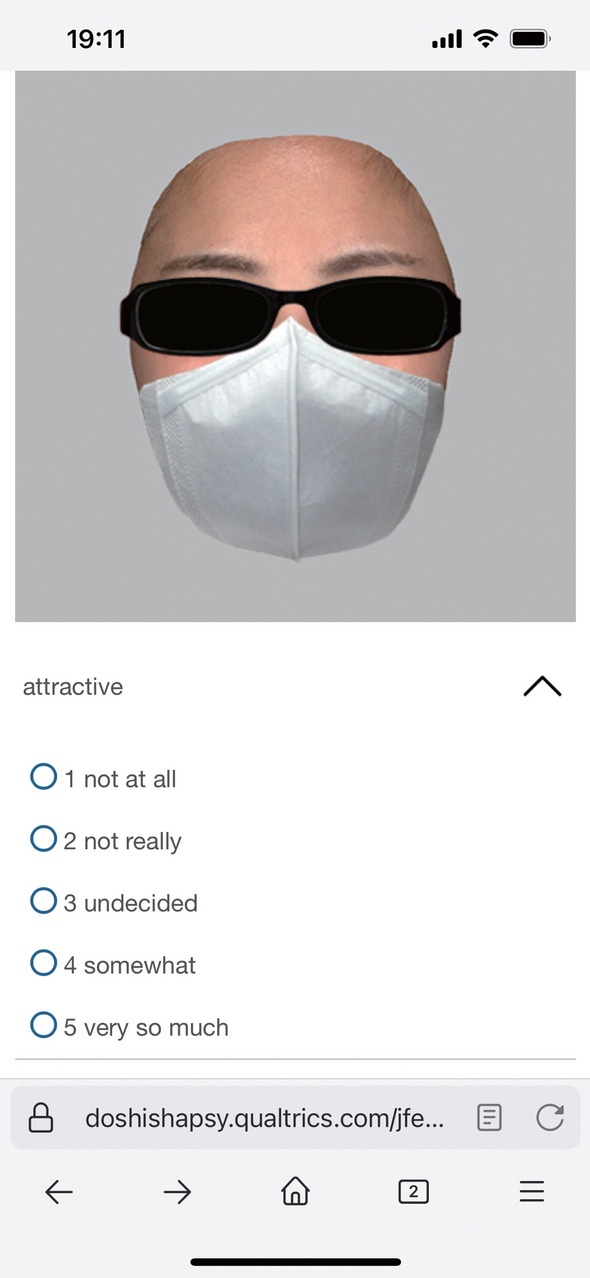
A screenshot of experiment using Qualtrics on a smartphone

Both the Former and Latter experiments were conducted online using Qualtrics. We provided participants with the entrance URL of Qualtrics and asked them to start the experiment in an environment where they could access Qualtrics, such as a smartphone or tablets. Forty facial stimuli were presented one at a time in random order, and participants were asked to rate them for attractiveness, trustworthiness, and familiarity on 5-point Likert-type scales (Fig. 2). The rating was done at their own pace, and no feedback was given.

### Statistical analysis

The mean rating scores of each social trait (attractiveness, trustworthiness, and familiarity) were calculated in each combined condition of base attractiveness (HAF and LAF) and type of occlusion (no occlusion, face mask, sunglasses, and combination) for each participant. Then, rating data, which included attractiveness, trustworthiness, and familiarity, were individually compared using a three-way mixed-design analysis of variance (ANOVA), with a between-participant factor of time point and within-participant factors of base attractiveness and type of occlusion using a software “HAD” [42]. Follow-up simple interaction tests, simple-simple main effect tests, simple main effect tests, and multiple comparisons using Bonferroni-correction were conducted when necessary (*p* < 0.05).

## Results

Prior to presenting the results, the screen resolutions of the devices used by the participants are summarized in Table 1.

**Table 1:** Type and number of screen resolutions of devices used by participants

Former		Latter	
Resolution	Number of participants	Resolution	Number of participants
360 × 720	1	375 × 667	4
375 × 667	7	375 × 812	3
375 × 812	6	390 × 844	10
414 × 896	9	414 × 896	3
768 × 1024	1	820 × 1180	1
1280 × 720	7	834 × 1112	1
1366 × 768	1	834 × 1194	1
1368 × 912	2	1280 × 720	10
1440 × 900	5	1368 × 912	5
1536 × 864	7	1440 × 900	4
1600 × 900	1	1504 × 1003	2
1729 × 1120	1	1536 × 864	3
1824 × 1216	1	1536 × 1024	2
1920 × 1080	1	1920 × 1080	1

### Attractiveness

**Fig. 3:**
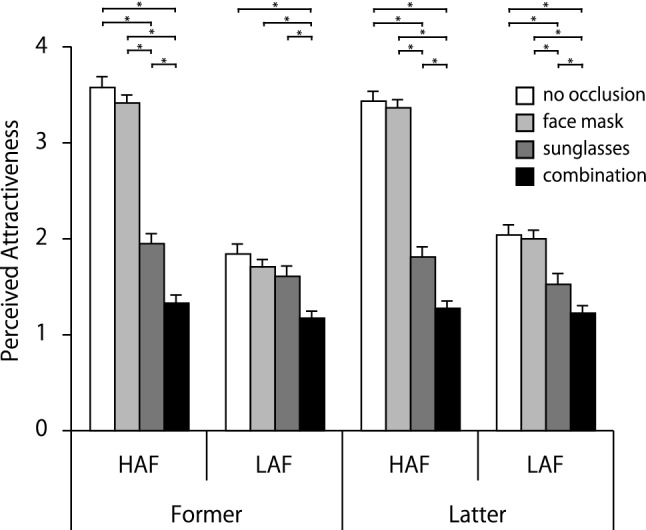
Mean attractiveness ratings for each condition. Error bars represent standard errors. Lines with * indicate that Bonferroni-corrected pairwise comparisons between conditions were significant, *p* < .05

Figure 3 shows the mean values of perceived attractiveness. A three-way interaction was significant, *F* (3, 294) = 2.89, *p* = 0.035, *η*_p_^2^ = 0.03. A simple interaction of time point × base attractiveness for no occlusion and face mask was significant, *F* (1, 98) = 6.15, *p* < 0.05, *η*_p_^2^ = 0.06; *F* (1, 98) = 6.01, *p* < 0.05, *η*_p_^2^ = 0.06, respectively. The simple interaction of base attractiveness × type of occlusion in Former and Latter was also significant, *F* (3, 294) = 178.73, *p* < 0.001, *η*_p_^2^ = 0.65; *F* (3, 294) = 121.78, *p* < 0.001, *η*_p_^2^ = 0.55, respectively. In contrast, the simple interaction of time point × type of occlusion was not significant. The simple-simple main effect of time point is significant in the LAF/face mask condition, *F* (1, 784) = 7.63, *p* < 0.01, *η*_p_^2^ = 0.07, indicating that Latter was more attractive than Former. The simple-simple main effect of base attractiveness was also significant, Former/no occlusion: *F* (1, 392) = 538.82, *p* < 0.001, *η*_p_^2^ = 0.92; Former/face mask: *F* (1, 392) = 519.22, *p* < 0.001, *η*_p_^2^ = 0.91; Latter/no occlusion: *F* (1, 392) = 341.29, *p* < 0.001, *η*_p_^2^ = 0.87; Latter/face mask: *F* (1, 392) = 327.66, *p* < 0.001, *η*_p_^2^ = 0.87. Additionally, simple main effects of base attractiveness for sunglasses and the combination conditions were significant, sunglasses: *F* (1, 392) = 33.61, *p* < 0.001, *η*_p_^2^ = 0.26; combination: *F* (1, 392) = 3.98, *p* < 0.05, *η*_p_^2^ = 0.04. These simple-simple and simple main effects of base attractiveness mean that HAFs are more attractive than LAFs, whether Former or Latter, and for any type of occlusion. The simple-simple main effect of type of occlusion was significant for Former/LAF and Latter/LAF, *F* (3, 588) = 23.82, *p* < 0.001, *η*_p_^2^ = 0.33; *F* (3, 588) = 44.01, *p* < 0.001, *η*_p_^2^ = 0.47, respectively. Multiple comparisons revealed that no occlusion and face mask were rated the most attractive in Latter/LAF, followed by sunglasses, but the combination was the least attractive. In contrast, no occlusion, face mask, and sunglasses were rated most attractive in Former/LAF, and combination was the least attractive in Former/LAF. Besides, the simple main effect of type of occlusion in HAF was significant, *F* (3, 588) = 683.64, *p* < 0.001, *η*_p_^2^ = 0.88. This means that regardless of time point, no occlusion and face mask were judged equally attractive in HAF, followed by sunglasses, with combination rated lowest.

### Trustworthiness

**Fig. 4:**
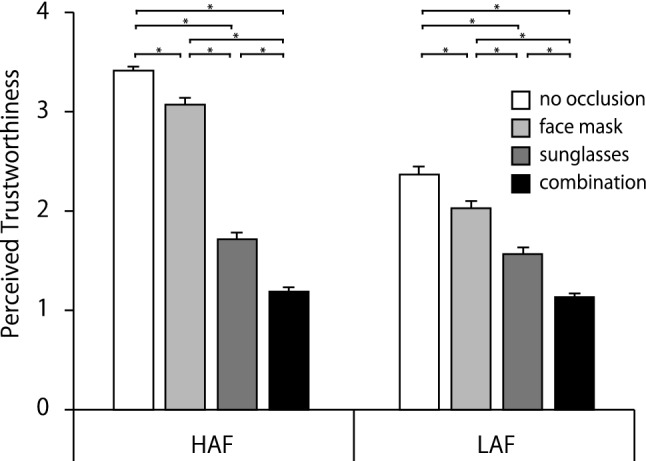
Mean trustworthiness ratings for each condition. Error bars represent standard errors. As the time effect was not significant, data were averaged across time point. Lines with * indicate that Bonferroni-corrected pairwise comparisons between conditions were significant, *p* < .05

Figure 4 shows the mean values of perceived trustworthiness. Two-way interactions, time point × base attractiveness and base attractiveness × type of occlusion, were significant, *F* (1, 98) = 4.87, *p* = 0.03, *η*_p_^2^ = 0.05; *F* (3, 294) = 151.77, *p* < 0.001, *η*_p_^2^ = 0.61, respectively. The simple main effects of time point on HAF and LAF conditions were not significant, indicating that trustworthiness did not vary over time. The simple main effects of base attractiveness in Former and Latter conditions were significant, *F* (1, 98) = 191.96, *p* < 0.001, *η*_p_^2^ = 0.80; *F* (1, 98) = 115.20, *p* < 0.001, *η*_p_^2^ = 0.70, respectively. Perceived trustworthiness was higher for HAFs than LAFs for both Former and Latter. Simple main effects of base attractiveness in the three types of occlusion conditions were also significant, no occlusion: *F* (1, 392) = 412.30, *p* < 0.001, *η*_p_^2^ = 0.81; face mask: *F* (1, 392) = 442.15, *p* < 0.001, *η*_p_^2^ = 0.82; sunglasses: *F* (1, 392) = 9.06, *p* < 0.01, *η*_p_^2^ = 0.09, respectively, indicating that HAFs had higher perceived trustworthiness than LAFs. The simple main effects of type of occlusion in HAF and LAF conditions were also significant, HAF: *F* (3, 588) = 547.36, *p* < 0.001, *η*_p_^2^ = 0.85; LAF: *F* (3, 588) = 143.00, *p* < 0.001, *η*_p_^2^ = 0.59, respectively. Multiple comparisons showed that for both HAF and LAF, no occlusion, face mask, sunglasses, and combination were more trustworthy, respectively.

### Familiarity

**Fig. 5:**
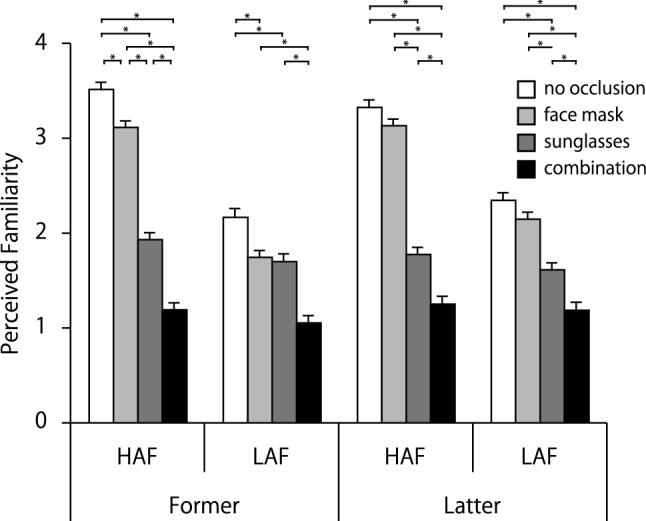
Mean familiarity ratings for each condition. Error bars represent standard errors. Lines with * indicate that Bonferroni-corrected pairwise comparisons between conditions were significant, *p* < .05

Figure 5 shows the mean values for perceived familiarity. For familiarity, a three-way interaction was significant, *F* (3, 294) = 3.56, *p* = 0.02, *η*_p_^2^ = 0.04. Although the simple interactions of time point × base attractiveness in no occlusion and face mask conditions were significant, *F* (1, 392) = 12.69, *p* < 0.001, *η*_p_^2^ = 0.12; *F* (1, 392) = 13.54, *p* < 0.001, *η*_p_^2^ = 0.12, respectively, not significant for sunglasses and combination conditions. The simple-simple main effect of time point was significant only for LAF/face mask, *F* (1, 784) = 12.31, *p* < 0.001, *η*_p_^2^ = 0.11, with Latter rated as more familiar than Former. Simple-simple main effects of base attractiveness were significant except for Former/combination and Latter/combination conditions, Former/no occlusion: *F* (1, 392) = 345.97, *p* < 0.001, *η*_p_^2^ = 0.88; Former/face mask: *F* (1, 392) = 354.25, *p* < 0.001, *η*_p_^2^ = 0.88; Former/sunglasses: *F* (1, 392) = 9.96, *p* < 0.01, *η*_p_^2^ = 0.17; Latter/no occlusion: *F* (1, 392) = 183.94, *p* < 0.001, *η*_p_^2^ = 0.79; Latter/face mask: *F* (1, 392) = 185.45, *p* < 0.001, *η*_p_^2^ = 0.79; Latter/sunglasses: *F* (1, 392) = 4.90, *p* = 0.027, *η*_p_^2^ = 0.09. This suggests that there was no difference in base attractiveness for either Former or Latter in combination conditions but that HAF was otherwise rated as more familiar than LAF. The simple-simple main effects of type of occlusion were significant under all conditions, Former/HAF: *F* (3, 588) = 252.04, *p* < 0.001, *η*_p_^2^ = 0.84; Former/LAF: *F* (3, 588) = 46.26, *p* < 0.001, *η*_p_^2^ = 0.49; Latter/HAF: *F* (3, 588) = 228.10, *p* < 0.001, *η*_p_^2^ = 0.82; Latter/LAF: *F* (3, 588) = 60.50, *p* < 0.001, *η*_p_^2^ = 0.55. Multiple comparisons showed that no occlusion, face mask, sunglasses, and the combination had the highest familiarity ratings in Former/HAF, respectively. Former/LAF showed a similar trend to Former/HAF, but there was no significant difference between face mask and sunglasses conditions. Additionally, both Latter/HAF and Latter/LAF had similar familiarity ratings for no occlusion and face mask conditions, followed by sunglasses and combination conditions, respectively.

## Discussion

Studies examining changes in perceived social traits using the paradigm of occluding the face have reported various effects. In studies examining the effects of face masks on social traits, which increased rapidly after the COVID-19 pandemic, the results were not always consistent, suggesting cultural differences. Few studies have examined the effects of sunglasses on social traits, and no study has explored changes in social traits due to both face masks and sunglasses over time, other than Bennetts et al. [28]. Therefore, in this study, we asked a Japanese sample to rate the attractiveness, trustworthiness, and familiarity of faces occluded by face masks and sunglasses at two time points, in late September 2020 and early April 2022, and compared the results.

### Hypothesis 1: Attractiveness

As in Kamatani et al. [14] using a Japanese sample, we predicted that perceived attractiveness would be higher for LAFs with face masks and lower for HAFs with face masks compared to unoccluded faces in the Former experiment. However, this prediction was not supported; there was no significant difference in perceived attractiveness between no occlusion and face mask conditions in either HAF or LAF conditions. This means that face masks already had little effect on attractiveness in Former in Japan, which is consistent with the results of Guo et al. [18] and Bennetts et al. [28]. Conversely, despite using the same Japanese faces as Kamatani et al. [14], one reason for the disagreement with their results is the property of the facial stimuli. The face stimuli used in the Kamatani et al. [14] experiment included hair, whereas that in this study were hair-trimmed. As Fink et al. [43] claimed that hair is a salient feature of human physical appearance, which contributes to the perception of beauty, the presence of hair may have affected the discrepancy in results. In addition, inconsistencies in the screen resolutions of the devices used by the participants may be one reason. As summarized in Table 1, the screen resolutions of the devices used by the participants in this experiment were not consistent. In contrast, the study by Kamatani et al. [14] was conducted in a laboratory setting with screens of the same size. Even though some studies reported that online experiments are acceptable [39–41], it is possible that the inconsistencies in screen resolution underlie the failure to replicate the results of Kamatani et al. [14]. Contrastingly, the mere exposure effect [38] was expected to increase perceived attractiveness in both HAF and LAF in Latter. As the simple-simple main effect of time point was significant only in LAFs wearing face masks, the prediction was supported only for LAFs wearing face masks. This may be a phenomenon specific to LAFs wearing face masks as perceived attractiveness in HAFs wearing face masks is sufficiently high.

We predicted that for both HAFs and LAFs, faces wearing sunglasses and faces wearing both sunglasses and face masks would have lower perceived attractiveness than unoccluded faces or faces wearing face masks in Former. We also predicted that there would be no time effect and that Latter would have similar results as Former. The results in Former showed that our predictions were supported except for LAFs wearing sunglasses. This is in disagreement with the results of Graham and Ritchie [27] and also suggests partial agreement with Bennetts et al. [28]. In the Bennetts et al. [28] study, faces wearing sunglasses and both face masks and sunglasses had lower perceived attractiveness, but there was no difference between them. Hence, they stated that it is difficult to determine whether the reduced attractiveness of faces wearing both face masks and sunglasses is because social cues from the face and facial symmetry are obscured or because those wearing both sunglasses and face masks are trying to hide their faces. Limited to the results of this study with a Japanese sample, the perceived attractiveness was higher for faces wearing sunglasses, which allow the lower half of the face to be seen, than for faces wearing both sunglasses and a face mask. Therefore, the difference in perceived attractiveness between faces wearing sunglasses and those wearing both a face mask and sunglasses may depend on the number of social cues obtained from the face. However, the results of the simple-simple main effect of time point showed that neither the LAFs nor the HAFs confirmed the prediction, as there was no observed time effect on faces wearing sunglasses or both sunglasses and a face mask. However, regarding the type of occlusion, LAFs in Former showed no significant difference between faces wearing face masks and sunglasses but for Latter, the perceived attractiveness was higher for faces wearing face masks than for sunglasses. Notably, this is due to the increased attractiveness ratings for LAFs wearing face masks described above, and thus, does not mean that wearing sunglasses decreased perceived attractiveness.

Based on the results of the simple-simple and simple main effects of base attractiveness, HAFs were rated as more attractive than LAFs, regardless of the time point of the experiment or the combination of occluding items worn. This means that the base attractiveness of the selected faces in the preliminary experiment was correct. Two other important implications are also included. The first is that HAFs are judged more attractive than LAFs when occluding either the upper or lower half of the face, suggesting that base attractiveness does not change no matter where we focus on the components of the face. In other words, the claim that the area around the eyes is important for attractiveness is correct [15, 44, 45]; furthermore, it is possible to judge base attractiveness only from clues from the area around the mouth. The second is that HAFs were judged more attractive than LAFs, even when they wore both face masks and sunglasses, which occluded most social cues from their faces. Although some studies have shown that eyebrows convey social cues about approachability and threat [46], data on attractiveness do not exist, and thus they do not provide clues for discussing the results of this study. A possible alternative is a facial contour. When both face masks and sunglasses are worn, the only determinant of social traits is the contour of the face. It is possible that HAFs were more symmetrical in their facial contours than LAFs because more symmetrical faces have higher perceived attractiveness [47–49].

### Hypothesis 2: Trustworthiness

We predicted that for both LAFs and HAFs in Former, faces wearing face masks would have higher perceived trustworthiness than unoccluded faces and hold at the same level or even higher in Latter. Results showed that regardless of base attractiveness, unoccluded faces had higher perceived trustworthiness than faces wearing face masks. The predictions were not supported, and the results of this study were inconsistent with those of many previous studies [18–21, 50, 51]. Additionally, the prediction that Japanese people wear face masks because conformity to social norms is a top priority [35] and that this behavior is linked to higher trustworthiness was not supported. Contrarily, the results of this study were consistent with research showing that unoccluded faces have higher perceived trustworthiness than faces wearing face masks [17]. The reason why our results were inconsistent with those of Nakayachi et al. [35], who also studied the same Japanese sample, is that their study was a survey on face mask-wearing behavior based on the assumed risk of infection with a virus and did not measure perceived trustworthiness by presenting faces wearing face masks, as we did in this study. In this experiment, which was conducted in the absence of any priming about the risk of infection with the virus, wearing face masks may have physically covered the contextual cues from the lower half of the face, creating a negative impact [17], and the perceived trustworthiness from the face simply may have been lowered. In any case, the effect of face mask wearing on perceived trustworthiness is a robust phenomenon in Japan, as there is no time effect.

We predicted that faces wearing sunglasses and both face masks and sunglasses would have lower perceived trustworthiness than unoccluded faces and faces wearing face masks in Former, and that this effect would be replicated in Latter. This prediction was supported and consistent with the results of several previous studies [27, 29, 30]. Additionally, the finding that faces wearing both a face mask and sunglasses had lower perceived trustworthiness than faces wearing only sunglasses is consistent with the results of Bennetts et al. [28]. Although wearing sunglasses is cool [25], we believe that the perceived trustworthiness of faces wearing sunglasses, which block the gaze, was reduced because the eyes play an important role in the perception of trustworthiness [27, 31]. As previously mentioned, faces wearing face masks also have lower perceived trustworthiness, but faces wearing sunglasses are even less trustworthy than faces wearing face masks, suggesting that the eye region has richer cues about trustworthiness than the mouth region. Furthermore, faces wearing both face masks and sunglasses would have the lowest trustworthiness as social cues and symmetry information from the facial features would be obscured, and they might be perceived as deliberately trying to hide their faces [28]. Additionally, culture-specific reasons may lie at the root of the diminished trustworthiness of wearing sunglasses. That reason is that wearing sunglasses (especially dark sunglasses) in Japan itself has a negative cultural and social impact. In Japanese culture, dark sunglasses are sometimes considered a symbol of antisocial forces (i.e., the mafia and yakuza), and dark sunglasses wearers are often perceived as threatening or suspicious. This is not a myth; Mackie [52] describes the association between sunglasses and yakuza members, and Kühne [53] also states that yakuza customarily wear dark sunglasses. As can be understood from these descriptions, dark sunglasses wearers in Japan are more likely to be perceived as having negative social characteristics, which may have reduced perceived trustworthiness. Additionally, wearing both sunglasses and a face mask was perceived as more suspicious and had the lowest perceived trustworthiness. As with face mask-wearing, the effects of wearing sunglasses and both sunglasses and face masks are robust over time.

The results of the simple main effect tests for base attractiveness showed that perceived trustworthiness was higher for HAFs than for LAFs for both Former and Latter. Similarly, perceived trustworthiness was higher for HAFs than LAFs for unoccluded faces, faces wearing face masks, and sunglasses. Interestingly, unless the face is occluded by both a face mask and sunglasses, HAFs are deemed more trustworthy based on visible facial features alone. These series of results may reflect a beauty-is-good bias. However, the effect of base attractiveness could not be confirmed on faces wearing both face masks and sunglasses. This implies that the beauty-is-good bias disappears when all facial features are covered up, and trustworthiness is less likely to be perceived from facial contours, unlike attractiveness.

### Hypothesis 3: Familiarity

We predicted that for both LAF and HAF conditions, faces wearing face masks would have higher perceived familiarity than unoccluded faces in Former and hold at the same level or even increase in Latter. Regardless of base attractiveness, perceived familiarity was higher for unoccluded faces than for faces wearing face masks in Former, suggesting that the prediction was not supported. The results were also inconsistent with previous studies showing that faces wearing face masks were more familiar [18–21]. Contrary to these previous studies, Seres et al. [54] measured inter-personal distance in real-life outdoor waiting lines and found that persons kept greater distances to a confederate with a face mask compared to one without a face mask. According to them, participants associated face masks with a person’s wish for greater interpersonal distance and thus increased distances due to politeness. As Seres et al. [54] conducted a field experiment in Berlin during the first lockdown in Germany (April 2020), a direct comparison with this study may be difficult. However, this study’s results in Former are consistent with their idea that face masks signal to others that they should adopt stricter precautions. In other words, in early pandemic Japan, when a stranger wears a face mask, it means that he or she is not letting down his or her guard. In Latter, however, there was no significant difference in perceived familiarity between unoccluded and faces wearing face masks in either LAFs or HAFs. Although the year 2022 is when countries worldwide begin to make political decisions that wearing masks is unnecessary, Japanese people kept wearing face masks even when they knew they were not infected with COVID-19 to comply with social norms [35]. This social conformity may have eliminated the significant difference in familiarity that existed between the unoccluded faces and faces wearing face masks [18]. Alternatively, faces wearing face masks may have been a sign of health [14], and the degree of familiarity may have been equal between the unoccluded and faces wearing face masks [19, 21, 55]. In any case, face masks are nowadays becoming a social tool [56]. Notably, however, the significant simple-simple main effect of time point was only for LAFs wearing face masks. In other words, the time effect in familiarity is limited to LAFs wearing face masks. The perceived familiarity of HAF unoccluded faces in Former was higher than that of faces wearing face masks, and the familiarity of both in Latter was similar. This result suggests that the perceived familiarity of the faces wearing face masks increased and that there appears to be a time effect for HAFs. However, the apparent increase in familiarity is not a time effect as the simple-simple main effect of time point was not significant in the unoccluded HAFs and HAFs wearing face masks.

We predicted that for both LAFs and HAFs, faces wearing sunglasses and faces wearing both sunglasses and face masks would have lower perceived familiarity at any time point compared to unoccluded faces and faces wearing face masks. This prediction was largely supported. Similar to perceived trustworthiness, sunglasses wearers in Japan are more likely to be perceived as having negative social characteristics, which may have reduced perceived familiarity. Additionally, as Qiu et al. [57] noted that as eye contact in social interactions is linked to familiarity, sunglasses occluding eyes may have reduced perceived familiarity. However, wearing both sunglasses and a face mask was perceived as more suspicious and would have resulted in the lowest perceived familiarity. When the face was occluded with face masks and sunglasses, all information from the facial features was blocked, making it impossible to judge whether the person was familiar, and thus, the lowest rating was given.

The results of the simple-simple main effect tests of base attractiveness showed that perceived familiarity was higher for HAFs than for LAFs in both Former and Latter, except for faces wearing both face masks and sunglasses. Interestingly, as with perceived trustworthiness, HAFs are judged more familiar based on visible facial features alone unless the face is covered by both face masks and sunglasses, suggesting the possibility of a beauty-is-good bias in familiarity as well. Similar to perceived trustworthiness, the beauty-is-good bias disappears when all facial features are covered up, and familiarity cannot be perceived from facial contours.

## Conclusion

In perceived attractiveness, there was no significant difference between unoccluded faces and faces with face masks for either Former or Latter, with the lowest for faces wearing both sunglasses and face masks. The predicted time effect was limited, with Latter having higher perceived attractiveness than Former only for LAFs wearing face masks. In terms of perceived trustworthiness, regardless of time point and base attractiveness, trustworthiness was consistently higher for unoccluded faces, faces wearing a face mask, sunglasses, and both sunglasses and a face mask, respectively. Moreover, no time effect occurred. In perceived familiarity, similar to attractiveness, there was no significant difference between unoccluded faces and faces wearing face masks in Latter, and faces wearing sunglasses or both sunglasses and face masks were rated lower. As with attractiveness, the time effect was limited, with Latter having higher perceived familiarity than Former only for LAFs wearing face masks. In conclusion, social traits conveyed by faces were most positively received when the face was unoccluded in Japan. Recently, social traits of faces wearing face masks have also been found to be at a level similar to that of unoccluded faces. However, wearing sunglasses or both sunglasses and a face mask has a negative impact and should be avoided when establishing good interpersonal relationships.

Two primarily limitations exist in this study. The first is the issue of perceived familiarity with online experiments. Studies have shown that online experiments can be used to measure perceived attractiveness and trustworthiness from faces [39–41]. However, even with the existence of the beauty-is-good bias, it is not yet clear whether online experiments can accurately measure perceived familiarity. Second, we did not measure base ratings of perceived trustworthiness and familiarity in our preliminary experiment. However, studies have reported that differences in social traits and facial expressions change the effects of wearing a face mask [58, 59]. That is, this suggests that the results may differ depending on base ratings of perceived trustworthiness and familiarity.

Currently, the COVID-19 pandemic is coming to an end, and wearing face masks is no longer necessary in many countries. What kind of world will we be living in when we return to the pre-pandemic environment? Will face masks return to being a symbol of unhealthiness, as they were before, or will they continue to be recognized as part of the face? Will the impact of occluding the face with sunglasses change? Additionally, how will the cognitive mechanisms of social traits of children who spent their childhoods during the COVID-19 pandemic, when wearing face masks was common, develop as they grow up? Thus, it will be necessary to continue to test the effectiveness of face occluding on an ongoing basis, as various important issues lie in front of us that cannot be solved unless the future comes.

## Data Availability

The rating data for attractiveness, trustworthiness, and familiarity are available via the Centre Open Science repository, https://osf.io/2g4za/.
